# COVID-19-Associated Large- and Medium-Sized-Vessel Pathology: A Case Series

**DOI:** 10.1055/s-0042-1748960

**Published:** 2022-11-01

**Authors:** Stacey Chen, Jad Malas, Larry A. Latson, Navneet Narula, Amy V. Rapkiewicz, David M. Williams, Harvey I. Pass, Aubrey C. Galloway, Deane E. Smith

**Affiliations:** 1 Department of Cardiothoracic Surgery, New York University Langone Health, New York, New York; 2 Department of Radiology, New York University Langone Health, New York, New York; 3 Department of Pathology, New York University Langone Health, New York, New York

**Keywords:** COVID-19, aortitis, large-vessel vasculitis, medium-vessel vasculitis

## Abstract

**Background** Coronavirus disease-19 (COVID-19) remains a public health crisis. The epidemiology of COVID-19-associated large- and medium-sized-vessel pathology is not well characterized. The aim of this study is to identify patients with possible COVID-19-associated large- and medium-sized-vessel pathology based on computed tomography (CT) imaging to provide insight into this rare, but potentially devastating, cardiovascular manifestation.

**Methods** This is a single-center retrospective review of patients with CT chest, abdomen, and/or pelvis concerning for large- and medium-vessel pathology and confirmed COVID-19 infection from March 1, 2020 to October 31, 2020.

**Results** During the study period, 6,553 CT reports were reviewed and pertinent imaging was identified in 139 patients. Of these, 8 patients (median age: 59 years, range 51–82) were COVID-19 positive. All patients had preexisting cardiovascular risk factors and three (37.5%) had an autoimmune disease. Four patients were never hospitalized for COVID-19. Among these, two presented to the hospital at a median of 39 days (range: 27–50) after their initial COVID-19 test with chest and back pain where imaging revealed extensive aortic pathology. One patient required surgical management for aortic pathology. All other patients were treated with expectant management and outpatient follow-up.

**Conclusion** The clinical and radiological presentations of COVID-19-associated large- and medium-vessel pathology are heterogeneous and can be a late finding after COVID-19 recovery. Close clinical follow-up and surveillance imaging for large- and medium-sized-vessel pathology may be warranted in COVID-19 patients.

## Introduction

Coronavirus disease-19 (COVID-19) caused by the novel severe acute respiratory syndrome coronavirus-2 (SARS-CoV-2) remains an ongoing global pandemic with a total of 94 million reported infections and over 2 million deaths worldwide as of January 16, 2021. 1 While COVID-19 is primarily a respiratory illness, it can present with a wide range of clinical manifestations from asymptomatic infection to multiorgan system failure. 2 3 Studies have demonstrated that endothelial cell dysfunction in the setting of SARS-CoV-2 infection plays an important role in the pathophysiology of the activation of the innate and adaptive immune systems that can result in an overwhelming systemic proinflammatory response with venous and arterial thromboembolic phenomena, endothelialitis, and vasculitis. 4 5

Increasing clinical experience and research have highlighted that COVID-19-associated cardiovascular complications are not only challenging, but may ultimately be devastating. The cardiovascular effects of SARS-CoV-2 are not well understood and remain an area of ongoing investigation. There is a growing body of literature detailing a new condition in the pediatric population associated with COVID-19 termed multisystem inflammatory syndrome in children (MIS-C), which has been described as a Kawasaki-like illness. 6 Similar to Kawasaki disease, MIS-C results in vasculitis of medium-sized vessels, notably aneurysmal formation of the coronary artery, which has been reported to occur at an incidence of 14 to 48%. 7 There are now emerging reports of large-vessel vasculitis in pediatric and adult patients in association with COVID-19. 8 9 10 A better understanding of the incidence of COVID-19-associated large- and medium-sized-vessel vasculitis is important to guide clinician decision-making regarding treatment and surveillance imaging. The purpose of this study was to identify patients with COVID-19-associated large- and medium-sized vessel pathologies based on computed tomography (CT) imaging and review their baseline characteristics, COVID-19 clinical course, and outcomes.

## Methods

A retrospective search of the picture archiving and communication system was conducted for all CT examinations (i.e., without contrast, with contrast, and angiogram) involving the chest, abdomen, and/or pelvis in inpatient and outpatient adults ≥18 years old from March 1, 2020 to October 31, 2020 at New York University Langone Health (NYULH). The search terms “aortitis,” “penetrating ulcer,” “vasculitis,” “dissection,” “intramural hematoma,” and “pseudoaneurysm” were used. A total of 6,553 reports including at least one of these terms were identified. The full text of all imaging reports from the search was screened by a cardiovascular radiologist for positive findings of vascular pathology. Because large- and medium-sized-vessel vasculitis are general terms used to describe a heterogeneous group of infectious and noninfectious diseases, a broad definition was used to screen for positive CT findings. Large vessels were defined as involvement of the aorta and/or its major branches. CT images that qualified for positive vascular pathology included abnormal wall thickening surrounding a vessel lumen, periaortic wall thickening with or without associated fusiform vascular dilatation, and surrounding fat and/or soft tissue changes.

Of the 6,553 reports, 139 relevant CT images were identified. Patients with positive radiologic findings were cross-referenced with review of their respective electronic medical records for a diagnosis of COVID-19, which was confirmed by a positive result on reverse transcriptase polymerase chain reaction (RT-PCR) assay of a specimen collected by nasopharyngeal swab. A total of eight patients with COVID-19 and pertinent imaging on CT were identified and their data were collected from direct electronic medical record review. This study was approved by the institutional review board at NYULH and because of its retrospective nature, informed patient consent was waived.

## Results

### Study Population

From March 1, 2020 to October 31, 2020, eight patients (median age: 59 years, range: 51–82) with confirmed COVID-19 by RT-PCR and CT imaging concerning for large- and medium-vessel pathology were identified. Patient demographics and clinical and laboratory information are presented in Table 1 . No patients had any known preexisting aortic pathology or history of connective tissue disorders. All patients had at least one cardiovascular risk factor—six (75%) had hypertension and four (50%) had a smoking history. Three patients (37.5%) had a known autoimmune disease and none were on steroid therapy.

**Table 1:** Baseline characteristics and relevant clinical and laboratory information in patients with COVID-19-associated large-vessel pathology

Patient number	1	2	3	4	5	6	7	8
Age, y	59	52	59	59	75	82	71	56
Gender	F	F	M	F	F	M	M	M
Body mass index, kg/m ^2^	29.28	28.32	28.12	24.14	30.74	30.62	48.94	N/A
Diabetes	No	No	No	No	Yes	No	No	No
Hypertension	Yes	No	Yes	Yes	Yes	Yes	No	Yes
Coronary artery disease	No	No	No	Yes	No	No	No	No
Chronic kidney disease	No	No	No	No	Yes	Yes	No	Yes
Peripheral vascular disease	No	No	No	No	No	Yes	No	No
Smoking history	Never smoker	Current smoker	Former smoker	Never smoker	Never smoker	Former smoker	Never smoker	Unknown
Preexisting aortic pathology	No	No	No	No	No	No	No	No
Chronic obstructive pulmonary disease	No	No	No	No	No	Yes	No	No
Asthma	No	Yes	No	No	No	No	Yes	No
Autoimmune disease	No	Hashimoto's thyroiditis	Graves' disease	Multiple sclerosis	No	No	No	No
Management setting of COVID-19	Outpatient	Outpatient	Outpatient	Inpatient (ICU)	Inpatient (non-ICU)	Outpatient	Inpatient (ICU)	Inpatient (ICU)
COVID-19 disease severity	Mild	Mild	Mild	Critical	Moderate	Mild	Moderate	Unable to determine a
Symptoms on admission								
Fever/Chills	No	No	N/A	Yes	No	No	No	No
Cough	No	No	N/A	No	Yes	No	No	No
Malaise	No	No	N/A	Yes	Yes	No	No	No
Dyspnea	No	No	N/A	No	Yes	No	Yes	No
GI distress	No	No	N/A	Yes	No	No	No	No
Chest pain	Yes	Yes	N/A	No	No	No	Yes	Yes
Back pain	Yes	Yes	N/A	No	No	No	No	No
Laboratory markers on admission								
WBC (10 ^3^ /µL)	11.9	7.2	N/A	18.3	10.9	5.6	11.3	14.4
Hemoglobin (g/dL)	9.1	13.1	N/A	10.5	12.1	11.4	14.5	13.1
Platelet (10 ^3^ /µL)	605	198	N/A	367	473	148	289	309
Creatinine (mg/dL)	0.86	0.79	N/A	4.4	0.83	1.44	1.35	1.5
AST (IU/L)	35	28	N/A	1480	29	N/A	34	35
ALT (IU/L)	45	40	N/A	1327	46	N/A	24	27
Total bilirubin (mg/dL)	0.3	0.3	N/A	0.6	0.5	N/A	0.6	0.3
PT (s)	15.0	12.6	N/A	15.8	13.4	12.7	17.0	13.7
aPTT (s)	32.2	32.5	N/A	28.5	38.6	29.7	59.8	33.0
CRP (mg/L)	220.1	<2.9	N/A	138.86	31.0	N/A	109.0	192.82
D-dimer (ng/mL)	1258	231	N/A	7454	974	N/A	432	11272
ESR (mm/h)	N/A	120	N/A	35	75	N/A	106	47
Ferritin (ng/mL)	1035	132.4	N/A	12978	770.9	N/A	185.3	245
LDH (U/L)	290	119	N/A	3827	278	N/A	296	247
Procalcitonin (ng/mL)	0.1	N/A	N/A	1.15	0.05	N/A	0.05	1.82
Immunologic and infectious laboratory tests								
Antinuclear antibody	Negative	Positive	Negative	N/A	N/A	N/A	N/A	N/A
HIV	Negative	Negative	Negative	N/A	N/A	N/A	N/A	N/A
RPR	Non-reactive	Non-reactive	Negative	N/A	N/A	N/A	N/A	N/A
Antiphospholipid syndrome antibodies	Negative	N/A	Negative	N/A	N/A	N/A	N/A	N/A
Blood culture	Negative	Negative	N/A	Positive	N/A	N/A	Negative	Negative
COVID-19 RT-PCR test b	Negative	Positive	Negative	Positive	Positive	Negative	Positive	Positive
COVID-19 IgG antibody (units) c	16.9	Not performed	6.7	Not performed	Not performed	Not performed	24.3	Not performed

Abbreviations: ALT, alanine transaminase; aPTT, activatedpartial thromboplastin time; AST, aspartate transaminase; COVID-19, coronavirus disease 2019; CRP, C-reactive protein; ESR, erythrocyte sedimentation rate; GI, gastrointestinal; HIV, human immunodeficiency virus; ICU, intensive care unit; LDH, lactate dehydrogenase; N/A, not available; No., number; PT, prothrombin time; RPR, rapid plasmin reagin; RT-PCR, reverse transcription polymerase chain reaction, IgG, immunoglobulin G; WBC, white blood cell count.

a Patient number 8 presented with cerebrovascular accident with acute deterioration in mental status, unable to determine clinical severity of COVID-19.

b COVID-19 RT-PCR test at time of imaging findings.

c IgG antibody testing at time of imaging findings.

### Clinical Presentation and Outcomes

Among the eight patients, four (50%) were not hospitalized and four (50%) were hospitalized for COVID-19. Of the patients who were hospitalized for COVID-19, all CT images were performed during admission. The indication for CT included assessment of pulmonary embolism (PE) in twopatients, abdominal pain in one patient, and to further evaluate an incidental finding of an ascending aortic dissection visualized on noncontrast head CT in one patient. Patients' COVID-19 treatments and overall outcomes are described in Table 2 .

**Table 2:** Relevant COVID-19 treatment and overall outcomes in patients with COVID-19 infection and large-vessel pathology

Patient number	**1**	**2**	**3**	**4**	**5**	**6**	**7**	**8**
COVID-19 treatment								
Hydroxychloroquine	No	No	No	No	Yes	No	No	Yes
Tocilizumab	No	No	No	No	Yes	No	No	No
Azithromycin	No	No	Yes	No	No	No	Yes	Yes
Antibiotics	No	No	No	Yes	Yes	No	Yes	Yes
Steroids	No	No	No	Yes	Yes	No	Yes	No
Ascorbic acid	No	No	No	No	Yes	No	No	No
Cholecalciferol	No	No	No	No	Yes	No	No	No
Zinc sulfate	No	No	No	No	No	No	No	Yes
Anticoagulation	No	No	No	No	Yes	No	Yes	No
Vascular pathology imaging	Aortitis with multifocal pseudoaneurysms of thoracic aorta	Aortitis of distal thoracic aorta extending to proximal abdominal aorta	Segmental aortitis with rind of soft tissue thickening around infrarenal abdominal aorta	Vasculitis with diminished caliber of celiac trunk branch vessels, superior mesenteric artery, and renal arteries	Penetrating ulcer at distal aortic arch	Penetrating ulcer at proximal aortic arch	Right coronary artery aneurysm	Type A thoracoabdominal aortic dissection
Vascular pathology treatment	Surgery	Medical (Steroids)	None	None	None	None	None	None
Vascular pathology follow-up imaging	Resolved	Resolved	Persistent	N/A	Unknown	Unknown	Unknown	N/A
Overall outcome	Alive	Alive	Alive	Expired	Alive	Alive	Alive	Expired

Abbreviations: COVID-19, coronavirus disease 2019; N/A, not available.

Three patients (37.5%; patient numbers 1–3) had imaging concerning for large-vessel vasculitis, specifically aortitis, of COVID-19. Patients number 1 and 2 developed large-vessel vasculitis-associated symptoms and presented to the hospital at a median of 39 days (range: 27–50) after their initial COVID-19 test with chest and back pain where diagnostic CT demonstrated aortitis. At the time of presentation, both patients were hemodynamically stable and admitted to the hospital for treatment—one patient required surgical intervention while the other was treated medically.

**Fig. 1:**
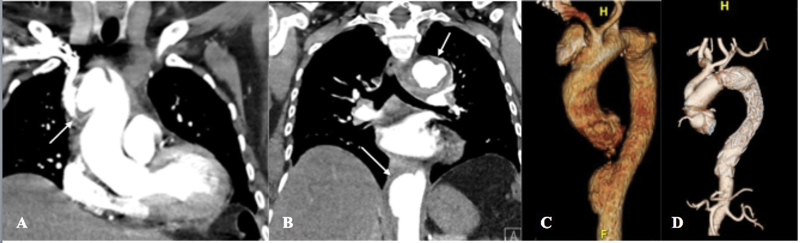
( **A** ) Admission computed tomography angiogram (CTA; coronal view) of patient 1 demonstrating 4.5 cm saccular pseudoaneurysm of distal ascending aorta and proximal aortic arch (arrow). ( **B** ) CTA (coronal view) of patient 1 with descending thoracic aortic pseudoaneurysms (arrows). ( **C** ) Preoperative three-dimensional reconstruction of patient 1 demonstrates multiple contained aortic ruptures and bovine aortic arch. ( **D** ) Postoperative three-dimensional reconstruction of patient 1 with intact ascending and descending aortic aneurysm repairs.

Laboratory results in the surgical patient (patient number 1) confirmed previous COVID-19 infection with negative COVID-19 RT-PCR and positive COVID-19 immunoglobulin G (IgG) antibodies. The patient's CT angiogram (CTA) chest, abdomen, and pelvis demonstrated multiple contained ruptures in the thoracic aorta including a 4.5-cm saccular pseudoaneurysm of the distal ascending aorta and proximal aortic arch ( Fig. 1A – C ). This patient underwent urgent thoracic endovascular aortic repair of her descending thoracic aortic pseudoaneurysms followed by repair of her ascending aortic pseudoaneurysm with a zone II aortic arch technique. The patient made an uneventful recovery with intact neurologic function. Bacterial and fungal stains of the surgical specimen were negative and histopathology revealed patchy necrosis of the aortic wall with multiple sterile abscesses characterized by infiltrating neutrophils. We are currently analyzing this aorta with the GeoMx COVID-19 Immune Response Atlas probes (NanoString Technologies).

**Fig. 2:**
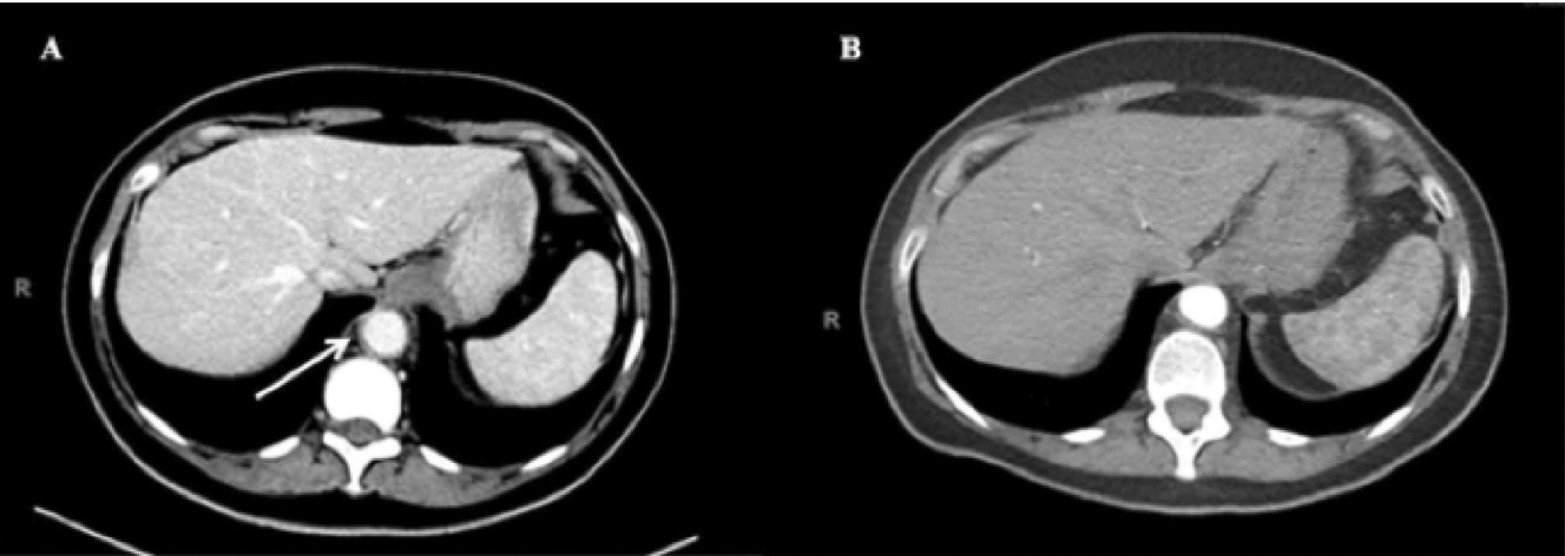
( **A** ) Admission computed tomography angiogram (CTA; axial view) of patient 2, demonstrating wall thickening and perivascular stranding of distal thoracic aorta without aneurysm (arrow). ( **B** ) Interval CTA (axial view) 3 months after patient 2 was treated with steroids without evidence of thoracic aortitis, aneurysm, or dissection.

In patient number 2 who was medically treated, laboratory results revealed persistent COVID-19 infection with positive COVID-19 RT-PCR. The patient's CTA chest, abdomen, and pelvis demonstrated aortitis involving the distal thoracic and proximal abdominal aorta extending to the origin of the celiac axis ( Fig. 2A ). This patient was started on pulse dose steroids and within 2 days, she reported complete resolution of symptoms. She was discharged home on hospital day 3 with a tapered steroid regimen.

During their hospitalization, both patients underwent infectious and immunologic workup for their aortitis, including antinuclear antibody (ANA), rapid plasma reagin, and antiphospholipid antibody panels, which were unremarkable.

Both patients underwent interval CTA following treatment 3 months later, which demonstrated intact ascending and descending thoracic aortic aneurysm repairs ( Fig. 1D ) in the surgical patient and resolution of aortitis in the medical patient ( Fig. 2B ). Both remain asymptomatic and will continue to undergo surveillance imaging.

**Fig. 3:**
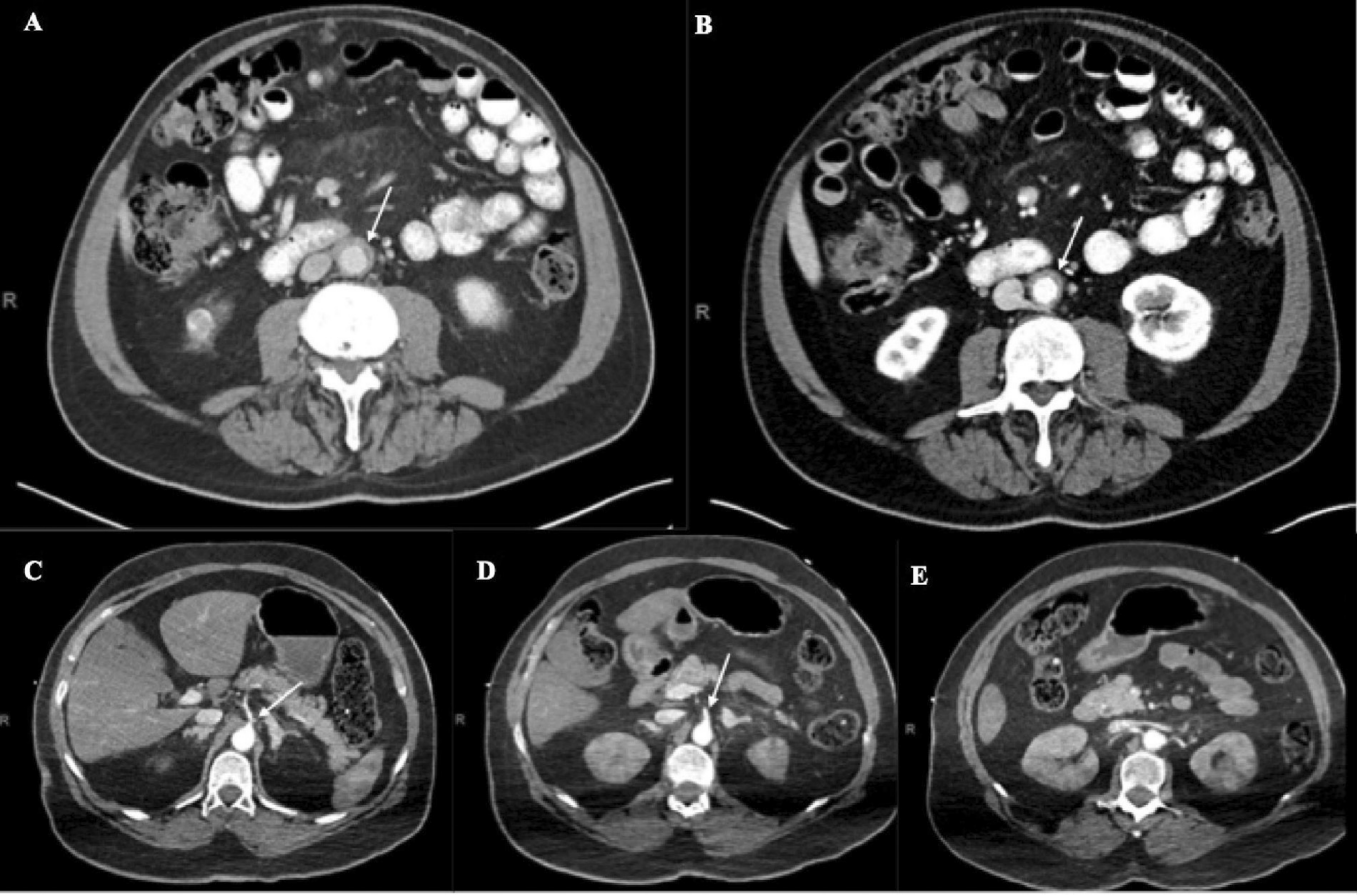
( **A** ) Computed tomography angiogram (CTA; axial view) of patient 3 demonstrating mild rind of soft tissue thickening surrounding infrarenal abdominal aorta (arrow). ( **B** ) Interval CTA (axial view) 3 months after ( **A** ) with unchanged rind of soft tissue thickening about the infrarenal abdominal aorta without aneurysm (arrow). ( **C** and **D** ) Admission CTA (axial view) of patient 4 demonstrating diminutive caliber of celiac trunk and superior mesenteric artery, respectively (arrow). ( **E** ) CTA (axial view) of patient 4 demonstrating hypoenhancement in bilateral kidneys.

In patient number 3, who never developed large-vessel vasculitis-associated symptoms, the pertinent radiographic finding was incidentally discovered on CT abdomen and pelvis with contrast for evaluation of new-onset pancreatic insufficiency and weight loss. Imaging demonstrated soft tissue thickening around the infrarenal abdominal aorta consistent with acute vasculitis ( Fig. 3A ). Based on these findings, the patient followed up with a rheumatologist and underwent COVID-19 IgG antibody testing, which was positive, and immunologic testing including ANA, antineutrophil cytoplasmic antibodies, and antiphospholipid syndrome antibody panel, which were unremarkable. An interval CT was performed 3 months later, which demonstrated an unchanged vasculitic process involving the infrarenal abdominal aorta ( Fig. 3B ). Given the patient's lack of symptoms, he was never treated and is currently undergoing further diagnostic workup.

Patient number 4 had imaging consistent with medium-sized-vessel vasculitis and was hospitalized for COVID-19. She presented to the emergency room (ER) with 2-week history of general malaise and worsening abdominal pain. The patient was hypothermic with body temperature of 35.5°C, hypotensive with mottled extremities, tachycardic with heart rate of 149 beats per minute, and tachypneic with respiratory rate of 30 breaths per minute. Oxygen supplementation and empiric treatment for sepsis were initiated. Laboratory evaluation indicated severe metabolic acidosis (pH 7.10, bicarbonate 6.3, lactate 10.9), mild acute respiratory distress syndrome (ARDS) (PaO _2_ /FiO _2_ 241), leukocytosis (white blood cell 18.3 × 10 ^3^ /µL), and rhabdomyolysis (creatinine kinase 47658 IU/L). Given concern for septic shock in the setting of COVID-19 pneumonia in conjunction with an underlying intra-abdominal pathology, CTA chest, abdomen, and pelvis was performed, which revealed vasculitis with decreased caliber of the celiac trunk branch vessels, proximal superior mesenteric artery, and bilateral renal arteries ( Fig. 3C–E ). The patient was admitted to the intensive care unit. Her respiratory function rapidly declined and she was intubated and mechanically ventilated. However, despite maximal medical treatment, she expired on hospital day 1.

**Fig. 4:**
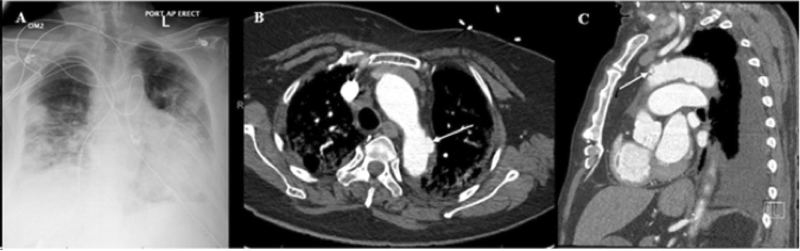
( **A** ) Admission anteroposterior chest radiograph of patient 5 demonstrating bilateral multifocal patchy airspace opacities and prominent interstitial markings. ( **B** ) Computed tomography angiogram (CTA; coronal view) of patient 5 demonstrating 1.5 cm × 0.8 cm outpouching at descending aortic arch compatible with penetrating ulcer (arrow). ( **C** ) CTA (sagittal view) of patient 6 demonstrating noncalcified ulcerated plaque in proximal aortic arch without intramural hematoma (arrow).

Two patients (25%) (patient numbers 5 and 6) had imaging concerning for penetrating aortic ulcer (PAU) involving the aortic arch. Patient number 5 presented to the ER with 4-day history of dyspnea, nonproductive cough, and general malaise. The patient was afebrile, tachycardic with heart rate of 114 beats per minute, and hypoxic with an oxygen saturation of 86% on room air. Chest radiograph (CXR) demonstrated bilateral multifocal airspace opacities with prominent interstitial markings ( Fig. 4A ). The patient was admitted to the hospital for COVID-19 pneumonia and developed acute respiratory failure on hospital day 7. Given her acute respiratory decompensation with concern for PE, the patient underwent CTA chest, which demonstrated PAU at the distal aortic arch ( Fig. 4B ). No medical or surgical treatment for her aortic pathology was performed. The patient recovered from COVID-19 and was discharged home on hospital day 39.

Patient number 6 was never hospitalized for COVID-19 and was recently diagnosed with severe aortic stenosis. As part of his preoperative evaluation for transcatheter aortic valve replacement (TAVR), he underwent CTA chest, abdomen, and pelvis, which revealed a linear flap in the proximal aortic arch suspicious for a previous penetrating ulcer ( Fig. 4C ). No additional imaging or laboratory tests were performed to address the CTA findings and the patient underwent elective TAVR and was discharged home on postoperative day 1. To date, neither patient has undergone COVID-19 IgG antibody testing or outpatient follow-up for their aortic pathologies.

**Fig. 5:**
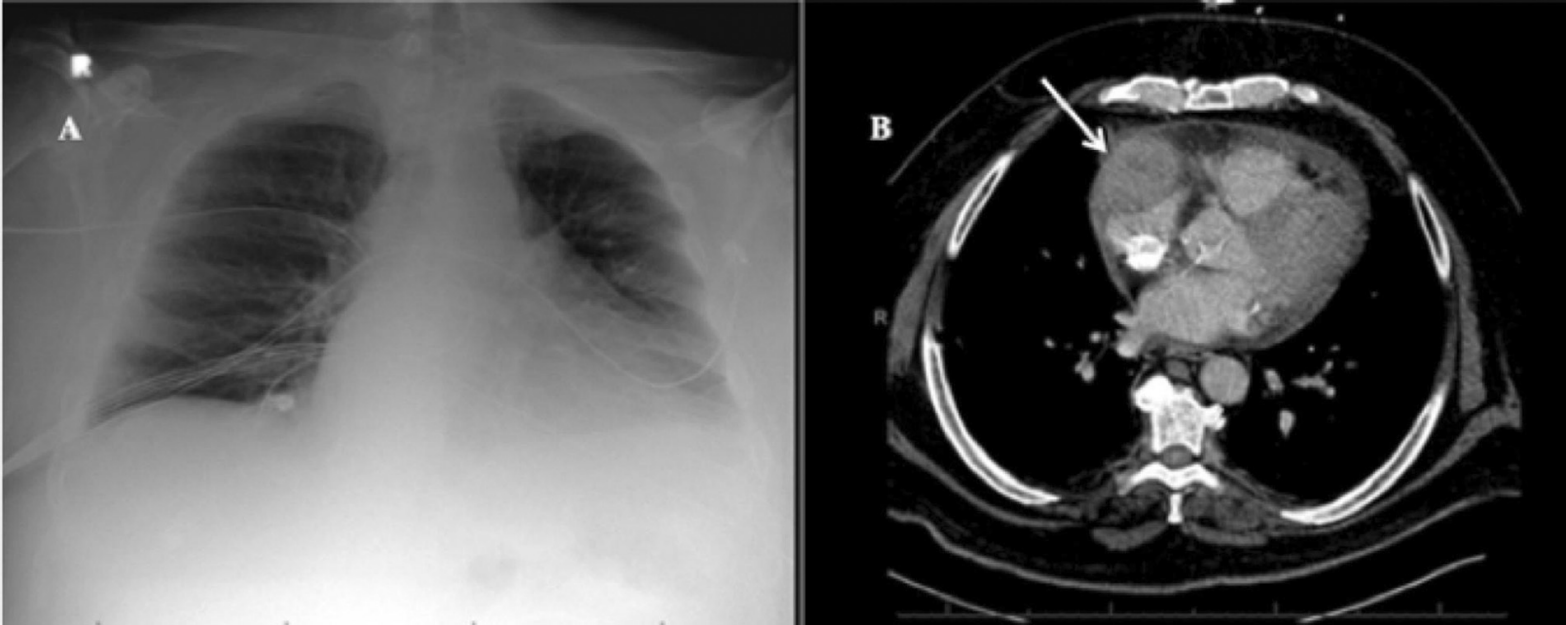
( **A** ) Admission anteroposterior chest radiograph of patient 7 with mild left basilar opacity. ( **B** ) Admission computed tomography angiogram (axial view) of patient 7 demonstrating 5.1 cm × 4.2 cm aneurysmal dilatation of right coronary artery with surrounding fluid and stranding as identified by the arrow.

Patient number 7 had imaging concerning for right coronary artery (RCA) aneurysm. The patient presented to the ER with 1-day history of left-sided pleuritic chest pain. He was afebrile with an oxygen saturation of 95% on room air;however, he became tachypneic with a respiratory rate of 28 breaths per minute. Initial evaluation was concerning for acute coronary syndrome with ST-segment elevations in the inferior leads on electrocardiogram and troponin of 5.35 ng/mL. Additional laboratory tests revealed positive COVID-19 RT-PCR and COVID-19 IgG antibodies. CXR demonstrated mild left basilar opacity ( Fig. 5A ). In the setting of COVID-19 infection, cardiac catheterization was initially deferred. Based on the patient's progressive tachypnea, he underwent CTA chest for suspected PE, which revealed a 5.1 cm × 4.2 cm RCA aneurysm ( Fig. 5B ) and he was admitted to the hospital. The patient's respiratory status remained stable and he underwent diagnostic cardiac catheterization on hospital day 3, which demonstrated chronic total occlusion of the proximal RCA with an associated large aneurysmal section. The patient recovered from COVID-19 and was discharged home. At his 1-week follow-up, the patient reported complete resolution of symptoms. Coronary artery bypass graft was recommended, however he declined surgery and has not presented for a follow-up appointment.

**Fig. 6:**
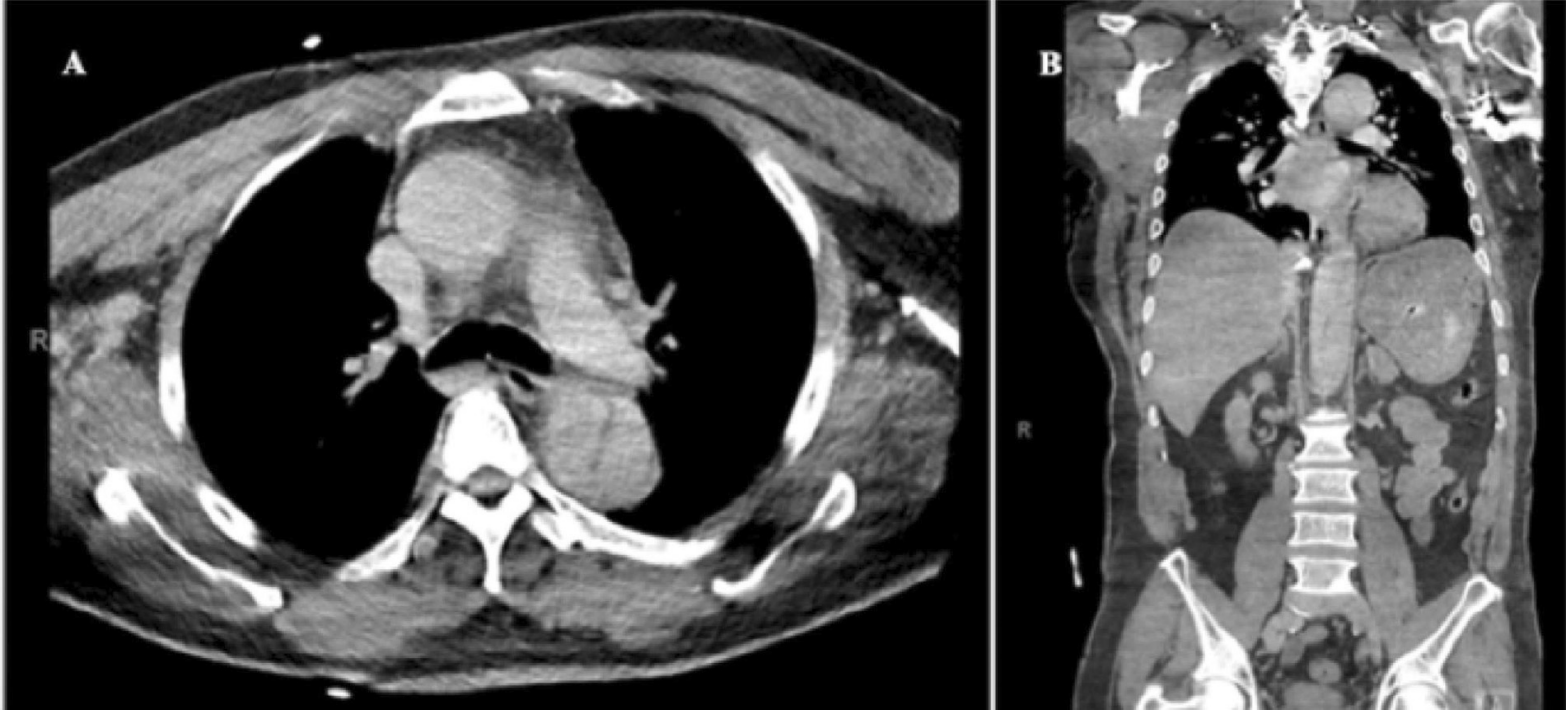
( **A** and **B** ) Admission computed tomography angiogram of patient 8 demonstrating axial and coronal view, respectively, of thoracoabdominal aortic dissection with ascending thoracic aorta dilation.

Patient number 8 presented with an acute aortic dissection. He was initially brought to the ER due to acute onset chest pain and subsequently developed left-sided hemiplegia, left facial droop, right gaze deviation, and dysarthria. The patient underwent noncontrast CT head that did not reveal any acute intracranial findings. A CTA head, neck, chest, abdomen, and pelvis was performed, which demonstrated a Stanford Type A aortic dissection beginning at the proximal aortic arch with extension into the proximal portion of the innominate artery and extending to the infrarenal aorta with associated hemopericardium ( Fig. 6A, B ). The patient became unresponsive and was intubated for airway protection. Laboratory tests indicated moderate ARDS (PaO _2_ /FiO _2_ 164) with D-dimer 11272 ng/mL. The patient was not offered surgery and he expired in the hospital. Postmortem evaluation revealed organizing thrombi with neutrophils within the area of the dissection. There was no evidence of aortitis.

## Discussion

COVID-19-associated large- and/or medium-sized-vessel vasculitis is rare. 8 9 10 The underlying mechanism for COVID-19-associated large- and medium-vessel pathology is most likely multifactorial and a combination of infectious and immunologic components. 11 The role of angiotensin-converting enzyme (ACE)-2 in cardiovascular disease and its distribution on the cell membranes of arterial and venous endothelia is well documented. 12 13 SARS-CoV-2 is characterized by its propensity to bind to ACE-2 and enter the endothelium of vessel beds, potentiating a systemic hyperinflammatory response that can result in viral vasculitis during the acute phase of the infection. 4 13 This pathophysiologic process can persist despite clinical recovery from the illness, which can result weeks later in a Type 3 hypersensitivity reaction with the deposition of antigen-antibody complexes in tissues, particularly blood vessels, with predominant neutrophilic infiltration as illustrated by Roncati et al. 14 This has been further supported in a radiologic study by Sollini et al 15 where 10 patients who recovered from COVID-19 but with unexplained persistent symptoms (i.e., dyspnea, fatigue, chest pain) for at least 30 days were imaged with [ ^18^ F]FDG positron emission tomography/CT and compared with propensity-matched controls to evaluate for radiologic findings that would explain these symptoms. They reported that although the total vascular score was similar between the two groups, the target-to-blood pool was higher in the thoracic aorta, right iliac artery, and femoral arteries of recovered COVID-19 patients, highlighting that COVID-19 induces a persistent systemic vasculitis despite illness recovery. 15

The clinical presentation and radiological findings in our case series varied greatly. The two patients with PAU on imaging may be less likely associated with COVID-19 as isolated PAUs have been reported to occur in 2.3 to 7.6% of acute aortic syndromes and typically present in older patients with cardiovascular risk factors. 16 However, several patients presented with unusual radiologic findings—2 with extensive aortitis, 1 with limited aortitis of the infrarenal aorta, 1 with a RCA aneurysm, and 1 with vasculitis of the splanchnic vessels. Three patients underwent rheumatologic and infectious evaluations which have been unremarkable and their aortic pathologies were not consistent with known infectious or noninfectious disorders. Notably, while COVID-19 IgG antibody testing was not performed in every patient, of the three patients who were tested for COVID-19 IgG, all three had positive antibodies. While its role in COVID-19 is not as well elucidated as Type 3 hypersensitivity reaction-mediated vasculitis, antibody-dependent enhancement (ADE) like that associated with Dengue fever has been postulated to enhance COVID-19 progression. 17 ADE is important to consider in the context of the coexistence of acute SARS-CoV-2 infection and COVID-19 IgG antibodies. 18 In a study of 26 patients with mild COVID-19, Wang et al 18 reported not only early IgG antibody detection within 7 days following confirmed COVID-19 by RT-PCR, but also long-term coexistence of IgG antibodies and active infection up to 50 days, demonstrating that early antibody production did not correlate with early virus elimination or recovery from disease. These findings suggest a possible underlying mechanism of viral enhancement of the adaptive immune system. Indeed, the patient who presented with an RCA aneurysm had coexisting IgG antibodies and COVID-19 infection. Thus, the pathologies described may in part be attributable to or exacerbated by an antibody-dependent response.

Definitive diagnosis of COVID-19-associated large- and medium-vessel pathology in our case series is limited by the lack of confirmatory histopathology. Histopathology reports were available for two patients and in the patient with aortitis there was a predominance of neutrophilic infiltration as seen with Type 3 hypersensitivity-mediated COVID-19 vasculitis. 14 The pathology of multiple sterile neutrophilic microabscesses in the patient with multiple contained thoracic aortic pseudoaneurysms was incongruent with the patient's clinical presentation and serologic results, suggesting COVID-19-associated aortitis. Conversely, in the patient with acute aortic dissection, it is possible that his COVID-19 infection accelerated his cardiovascular presentation. 19 It remains unclear if these are coincidental findings in COVID-19 patients as a result of CT imaging for other indications; however, the constellation of clinical, laboratory, and radiologic findings raises the clinical suspicion for COVID-19-associated vasculitis, suggesting that these pathologies represent a spectrum of early and late clinical sequelae of the disease and that patients with cardiovascular risk factors may be at increased risk. Interestingly, the degree of COVID-19 severity among patients did not correlate with aortic disease involvement on imaging as two of the most extensive cases of aortitis were found in patients who had mild COVID-19. Moreover, because we started our evaluation in patients with positive CT scans, this report most likely underestimates the number of patients with COVID-19-associated large- and medium-sized-vessel pathology at our institution. In the setting of COVID-19 with limited resources and a stringent goal toward infection control, inpatient CT imaging was limited to patients with high clinical suspicion for PEs or other acute intrathoracic or intra-abdominal pathologies. The heterogeneity of the clinical and radiologic presentations of the vascular pathologies in this case series illustrates the complexity of COVID-19 and suggests that further studies correlating COVID-19 IgG antibodies and disease progression as well as surveillance imaging for acute aortic pathology in COVID-19 patients should be an important consideration.

## Conclusion

COVID-19 remains a public health crisis. While significant advancements have been made regarding the medical management of this disease, the long-term ramifications of COVID-19 and its treatment remain to be seen. Thus far, we have identified several interesting vascular pathologies in patients with prior COVID-19 infection. It remains unclear how many recovered COVID-19 patients have undiagnosed large- and medium-sized vessel pathologies and the trajectory of their disease progression. We believe this association warrants further clinical and histopathologic investigation with a focus on identifying at-risk patients who would benefit from close surveillance.
